# The association between social media use and physical activity among Canadian adolescents: a Health Behaviour in School-aged Children (HBSC) study

**DOI:** 10.17269/s41997-023-00754-9

**Published:** 2023-03-15

**Authors:** Brandon Morningstar, Zahra Clayborne, Suzy L. Wong, Karen C. Roberts, Stephanie A. Prince, Geneviève Gariépy, Gary S. Goldfield, Ian Janssen, Justin J. Lang

**Affiliations:** 1 Centre for Surveillance and Applied Research, Public Health Agency of Canada, 785 Carling Ave, Ottawa, ON K1A 0K9 Canada; 2 School of Epidemiology and Public Health, Faculty of Medicine, University of Ottawa, Ottawa, ON Canada; 3 Department of Pediatrics, Cumming School of Medicine, University of Calgary, Calgary, AB Canada; 4 Centre for Health Promotion, Public Health Agency of Canada, Ottawa, ON Canada; 5 School of Public Health, University of Montreal, Montreal, QC Canada; 6 Healthy Active Living and Obesity Research Group, Children’s Hospital of Eastern Ontario Research Institute, Ottawa, ON Canada; 7 School of Kinesiology and Health Studies, Department of Public Health Sciences, Queen’s University, Kingston, ON Canada

**Keywords:** Exercise, Adolescent, Adolescent behaviour, Social media, Internet addiction disorder, Exercice, adolescent, comportement des adolescents, médias sociaux, trouble de dépendance à l’Internet

## Abstract

**Objective:**

To determine the association between social media use (SMU) and physical activity (PA) among Canadian adolescents.

**Methods:**

We used data from 12,358 participants in grades 6 to 10 who responded to the Canadian component of the 2017/2018 Health Behaviour in School-aged Children (HBSC) survey. Social media intensity and problematic SMU were assessed using a 4-point mutually exclusive scale that contained three categories based on intensity (non-active, active, and intense SMU) and one category based on the presence of addiction-like symptoms irrespective of intensity (problematic SMU). PA was assessed for five domains (i.e., school curriculum, organized sport, exercise, outdoor play, and active transport) and dichotomized using the first quartile to represent high PA engagement in each domain. Meeting PA recommendation of 60 min per day of moderate-to-vigorous PA was calculated using the sum of the five domains. Logistic regression models were used to assess the association between SMU and PA, with active SMU used as the reference group for all models.

**Results:**

Non-active SMU was associated with lower odds of meeting the daily PA recommendations and of high engagement in all five domains of PA when compared to active SMU. Intense SMU was associated with higher odds of meeting the daily PA recommendations. Problematic SMU was not associated with meeting daily PA recommendations, but it was significantly associated with lower odds of high PA engagement in the exercise domain.

**Conclusion:**

The findings of this study suggest that non-active SMU was significantly associated with lower PA levels. Problematic SMU was only significantly associated with lower PA levels in the exercise domain. Intense SMU was associated with higher odds of meeting the PA recommendation.

**Supplementary Information:**

The online version contains supplementary material available at 10.17269/s41997-023-00754-9.

## Résumé

**Objectif:**

Établir la relation entre l’utilisation des médias sociaux et l’activité physique chez les adolescents canadiens.

**Méthodes:**

Nous avons utilisé les données de 12 358 participants de la 6^e^ à la 10^e^ année qui ont répondu au volet canadien de l’Enquête sur les comportements de santé des jeunes d’âge scolaire de 2017–2018 (HBSC). L’intensité des médias sociaux et leur utilisation problématique ont été évaluées à l’aide d’une échelle à quatre points mutuellement exclusifs contenant trois catégories basées sur l’intensité (utilisation non active, active et intense des médias sociaux) et une catégorie basée sur la présence de symptômes de dépendance indépendamment de l’intensité (utilisation problématique des médias sociaux). L’activité physique a été évaluée pour cinq domaines (c’est-à-dire le programme scolaire, le sport organisé, l’exercice, le jeu en plein air et le transport actif) et dichotomisée en utilisant le premier quartile pour représenter la participation à une activité physique élevée dans chaque domaine. Le respect de la recommandation de 60 min par jour d’activité physique modérée à vigoureuse a été calculé en utilisant la somme des cinq domaines. Des modèles de régression logistique ont été utilisés pour évaluer la relation entre l’utilisation des médias sociaux et l’activité physique, l’utilisation active des médias sociaux étant utilisée comme groupe de référence pour tous les modèles.

**Résultats:**

L’utilisation non active des médias sociaux était associée à une probabilité plus faible de respecter les recommandations en matière d’activité physique quotidienne et à une participation élevée dans les cinq domaines de l’activité physique, par rapport à l’utilisation active des médias sociaux. Une utilisation intense des médias sociaux était associée à une probabilité plus élevée de respecter les recommandations en matière d’activité physique quotidienne. Une utilisation problématique des médias sociaux n’était pas associée au respect des recommandations en matière d’activité physique quotidienne, mais elle était fortement associée à une probabilité moindre de participation élevée à une activité physique dans le domaine de l’exercice.

**Conclusion:**

Les résultats de cette étude suggèrent que l’utilisation non active des médias sociaux est fortement associée à des niveaux d’activité physique plus faibles. Une utilisation problématique des médias sociaux n’était fortement associée à des niveaux d’activité physique plus faibles que dans le domaine de l’exercice. Une utilisation intense des médias sociaux était associée à une probabilité plus élevée de respecter la recommandation en matière d’activité physique.

## Introduction

The effects of excessive and problematic exposure to screen time on adolescent well-being, health, and development have previously been described in the literature (Lissak, 2018; Odgers et al., 2020; Przybylski & Weinstein, 2017). These studies investigated the association between the time spent on screens (e.g., phones or computers) and the negative effects on adolescent health and development (Lissak, 2018; Przybylski & Weinstein, 2017). However, recent studies have further scrutinized screen time use by how adolescents are interacting with screens, including what they contribute and gain from screen time use (Odgers et al., 2020). As such, different forms of screen time use, such as active, passive, educational, or social use, have been examined in the literature, specifically their effect on adolescent well-being and health behaviours (Sanders et al., 2019; Stiglic & Viner, 2019). With the growing presence and use of social media, there is concern surrounding its influence on adolescent well-being, health, and development (Uhls et al., 2017).

Social media can be defined as internet-based applications that facilitate the exchange of messages or user-generated content for the purpose of social connectedness, entertainment, or education (Kaess, 2020; Smahel et al., 2020). In 2017, approximately one third of Canadian adolescents reported using social media almost all the time throughout the day to communicate with others (Public Health Agency of Canada [PHAC], 2020). More recently, a study by Boniel-Nissim et al. (2022) characterized social media use with a combined scale that incorporated intensity (i.e., frequency) and problematic symptoms related to social media use. The previously validated social media use scale differentiated between non-active, active, intense, and problematic use, an approach that isolates problematic users regardless of intensity level (Boer et al., 2022). The differentiation between the presence of addiction-like symptoms (i.e., problematic social media use) and intensity of social media use is hypothesized to improve the mixed and conflicting research base on the association between social media and adolescent health and well-being (Boniel-Nissim et al., 2022).

With the increasing use of social media by adolescents, there is concern in regard to the extent it may have on all aspects of health, including health behaviours (Shimoga et al., 2019). Regular physical activity is important for adolescent development and health (Janssen & LeBlanc, 2010), although few Canadian youth attain the recommended amount of at least 60 min of daily moderate-to-vigorous intensity physical activity (MVPA) (Centre for Surveillance and Applied Research, 2021).

While not solely responsible, social media use may influence physical activity and overall health in a multidirectional manner. The relationship between social media use and physical activity is complex (Maher et al., 2016; Shimoga et al., 2019; Viner et al., 2019), with current literature outlining several theories. The first, and oldest theory, is the direct effect of time spent using social media displaces time away from physical activity (i.e., *the displacement theory*), displacing socializing, or by displacing or increasing other behaviours that can influence physical activity (e.g., sleep, school work, and sedentary time) (Hall & Liu, 2022; Viner et al., 2019). A second non-competing theory is that normative social media use is not detrimental to adolescent health and well-being. This has previously been described as the *digital Goldilocks theory*, wherein moderate social media use is not intrinsically harmful and overuse and underuse may limit the ability of adolescents to access social information or engage with peers (Boniel-Nissim et al., 2022; Przybylski & Weinstein, 2017). Going one step further, it is hypothesized that it is not necessarily overuse that is detrimental, but instead, it is problematic social media use (i.e., addiction-like symptoms toward social media) that negatively influences adolescent health (Boer et al., 2021, 2022; Boniel-Nissim et al., 2022).

While previous studies have investigated the association between social media use and physical activity (Rutter et al., 2021; Shimoga et al., 2019; Viner et al., 2019), none have done so using a social media use scale that includes a distinction between intensity and problematic use. Social media use has been previously defined as frequency of visiting different social media networks (Shimoga et al., 2019; Viner et al., 2019), frequency of checking and posting on social media networks (Rutter et al., 2021), or frequency of contact with others on social media (Boniel-Nissim et al., 2022). There is also a discrepancy scale used in some studies, where the highest reportable frequency ranges from “every day” (Shimoga et al., 2019) to “almost all the time throughout the day” (Boniel-Nissim et al., 2022) or “almost constantly” (Rutter et al., 2021). In these studies, there was no discernment between frequency of use and presence of symptoms of addiction-like symptoms, likely contributing to the conflicting and varying theories on the relationship between social media use and physical activity. Furthermore, applying the four-level scale of social media use exposure, as proposed by Boniel-Nissim et al. (2022), could provide an opportunity to test *the displacement and the digital Goldilocks theories* as they relate to physical activity.

As such, the objective of this study was to examine the association between the four-level exposure categories of social media use and meeting physical activity recommendations. The secondary objective was to assess the associations between social media use categories and engagement in physical activity across different domains (i.e., school curriculum, organized sport, exercise, outdoor play, and active transport). We hypothesized that both non-active and problematic social media use would be significantly and negatively associated with physical activity, as suggested by the digital Goldilocks theory.

## Methods

### HBSC Canada overview

This study used data from the Canadian component of the 2017/2018 Health Behaviour in School-aged Children (HBSC) survey. There are currently 51 participating countries or regions in North America and Europe. Participating countries or regions conducted the HBSC survey in their respective country or region using the same survey methods and questionnaire (Inchley et al., 2018). The Canadian HBSC is representative of approximately 93% of Canadian adolescents, with those in private or special schools, on First Nation or Inuit reservations, not in school, or incarcerated excluded from the study (PHAC, 2020). HBSC Canada used a random two-stage cluster sampling approach to recruit a nationally representative sample of students in grades 6 to 10 (typically ages 11 to 16). During the first stage, school jurisdictions were identified and clustered according to language of instruction, religious designation, and community size (PHAC, 2020). During the second stage, a list of eligible schools were identified in each cluster, and schools in these clusters were randomly selected to participate in the study. Recruitment and data collection for the Canadian HBSC study took place between January and May of 2018. The Public Health Agency of Canada (REB-2013–0022) and Queen’s University Research Ethics Board (6027003) granted ethics approval for the Canadian component of the HBSC. Participation in this study was voluntary and informed consent was obtained from school administrators, parents or guardians, and students following local protocols.

### Study sample

A total of 21,745 students from 287 schools participated in the Canadian component of the 2017/2018 cycle of the HBSC. Adolescents who identified gender as “neither term describes me” were excluded due to sample size (*n* = 336). An additional 9051 were excluded due to missing or incomplete entries. The majority of excluded entries were the result of incomplete or missing responses to the frequency of social media use and problematic social media use questions (*n* = 6087; 67.3%). A detailed comparison between excluded and included participants is available in Supplemental Table 1. These exclusions resulted in a final sample size of 12,358 students with complete information.

### Measures

#### Social media use intensity

Social media use intensity was assessed as previously described by Boniel-Nissim et al. (2022). Participants identified how often they had online contact with four different groups: their close friends, friends from a larger friend group, friends they met through the internet, and other people (i.e., parents, siblings, classmates, or teachers). For each group, participants reported how frequently they had online contact by selecting one of five frequency options ranging from “never or almost never” to “almost all the time throughout the day”. Students were then grouped to create a three-level social media use intensity scale based on the highest reported frequency of online contact across the four groups. Participants must have responded to the frequency of use for at least one of the four groups to be included in the analysis. Participants who reported “never or at most weekly” or “at least every week” were categorized as “non-active social media use”; students who reported “daily or almost daily”, or “several times each day” were categorized as “active social media use”; and students who reported “almost all the time throughout the day” were categorized as “intense” social media use.

#### Problematic social media use

Problematic social media use was assessed using the Social Media Disorder Scale (van den Eijnden et al., 2016). Participants were asked nine “yes” or “no” items pertaining to social media use that were related to addiction-like symptoms (e.g., preoccupation, tolerance, withdrawal, persistence, escape, problems, deception, displacement, and conflict). Participants must have complete responses for all nine questions to be included in the analysis. Those who responded “yes” to six or more of the items were categorized as problematic social media use.

#### Combined social media use scale

The problematic and social media use intensity categories were combined into four mutually exclusive categories, as previously described (Boniel-Nissim et al., 2022). These categories included non-active social media use (i.e., “non-active social media use” AND “no problematic use”), active social media use (i.e., “active social media use” AND “no problematic use”), intense social media use (i.e., “intense social media use” AND “no problematic use”), and problematic social media use (i.e., any category of the social media use intensity AND “problematic use”).

#### Physical activity

Five domains of physical activity were assessed. Physical activity was described in the survey as an activity that increases heart rate and causes breathlessness some of the time. Participants were asked how many hours a week they typically spent doing physical activity during class time at school (school curriculum domain; e.g., gym or fitness class), organized sports that were not part of classes (organized sport domain; e.g., school sports team or dance club), exercise sessions that were not part of gym class or organized sports (exercise domain; e.g., jogging, lifting weights, CrossFit), outdoor activities that were not part of gym class or organized sports (outdoor play domain; e.g., road hockey, skateboarding, active games at recess), and commuting by walking, cycling, or other active ways to get places (active transport domain; e.g., biking to school, walking to store). Corresponding time values (hours) were categorized into six response options: “none at all” = 0, “about half an hour a week” = 0.5, “about 1 hour a week” = 1, “about 2 hours a week” = 2, “about 3 hours a week” = 3, and “about 4 or more hours a week” = 4. These values were then multiplied by 60 and divided by 7 to convert to average minutes per day. Values across all five domains were combined to determine whether adolescents’ average daily MVPA met the daily MVPA recommendation of 60 min (Tremblay et al., 2016). Additionally, physical activity values for each domain were dichotomized using the first quartile to identify low (1st quartile) and high (2nd to 4th quartile) engagement.

#### Covariates

All covariates were collected using self-reported questionnaire responses. Covariates included gender (i.e., boy or girl), grade (i.e., grades 6–8 or 9–10), cultural and racial background (i.e., White, Black, Latin American, East and Southeast Asian, East Indian and South Asian, Arab and West Indian, and Other), time since immigration (i.e., Canadian born, 1 or 2 years, 3 + years), family affluence (i.e., low, medium, or high; Torsheim et al., 2016), and life satisfaction (i.e., low or high). All variables were predictors associated with physical activity or social media use (Kelly et al., 2018; Kukaswadia et al., 2014; Shimoga et al., 2019). For cultural and racial background, participants who identified as “Indigenous” were combined with the “Other (including mixed)” category to adhere to ethical restrictions with the HBSC data.

### Statistical analyses

The analysis was conducted using SAS Enterprise Guide version 7.1 (SAS, Cary, NC). All analyses incorporated survey weights to account for the distribution of adolescents in Canada. Descriptive analyses were performed on the full sample and by social media use category with 95% confidence intervals (CIs). A mixed effects logistic regression model clustered by school was used to test for differences in meeting the physical activity recommendation between social media use categories, with interaction terms included for grade and gender. A similar model was also used to test for engagement in physical activity for each physical activity domain by social media use categories. For all logistic regression analyses, active users served as the reference category as they were the most prevalent group in the sample.

## Results

Descriptive characteristics of the HBSC participants by social media use category are presented in Table 1. Overall, the most common social media use category was active use (*n* = 5468), followed by intense use (*n* = 4286), non-active use (*n* = 1768), and problematic use (*n* = 836).

**Table 1:** Descriptive characteristics for study participants by social media use category

Variable	Total sample (*n* = 12,358)	Non-active (*n* = 1768; 14.3%)	Active (*n* = 5468; 44.2%)	Intense (*n* = 4286; 34.6%)	Problematic (*n* = 836; 6.8%)
	Weighted % (95% CI)	Weighted % (95% CI)	Weighted % (95% CI)	Weighted % (95% CI)	Weighted % (95% CI)
Gender					
Boy	45.7 (43.7; 47.7)	56.9 (53.0; 60.8)^a^	46.3 (43.8; 48.9)	41.2 (38.4; 44.0)	36.4 (31.2; 41.7)^a^
Girl	54.3 (52.3; 56.3)	43.1 (39.2; 47.0)^a^	53.7 (51.1; 56.2)	58.8 (56.0; 61.6)	63.6 (58.3; 68.8)^a^
Grade					
6–8	58.2 (52.1; 64.2)	74.6 (69.2; 80.1)^a^	58.0 (51.9; 64.1)	51.5 (44.4; 58.7)	51.0 (42.6; 59.5)
9–10	41.8 (35.8; 47.9)	25.4 (19.9; 30.8)^a^	42.0 (35.9; 48.1)	48.5 (41.3; 55.6)	49.0 (40.5; 57.4)
Cultural and racial background					
White	71.6 (66.6; 76.5)	74.5 (68.6; 80.3)	74.1 (69.2; 79.0)	69.0 (63.9; 74.1)	59.9 (52.9; 67.0)^a^
Black	3.7 (2.3; 5.1)	4.2 (2.2; 6.2)	3.2 (1.9; 4.5)	4.0 (2.5; 5.5)	4.5 (1.8; 7.1)
Latin American	1.5 (1.0; 2.1)	1.0 (0.3; 1.7)	1.7 (0.9; 2.5)	1.5 (0.8; 2.3)	1.7 (0.3; 3.0)
East and Southeast Asian	3.2 (1.9; 4,5)	3.7 (1.8; 5.7)	3.3 (1.8; 4.9)	2.7 (1.4; 4.1)	2.8 (1.0; 4.5)
East Indian and South Asian	3.1 (1.8; 4.4)	2.6 (1.4; 3.8)	2.7 (1.5; 4.0)	3.6 (2.0; 5.3)	4.4 (0.3; 8.4)
Arab and West Indian	1.8 (1.1; 2.5)	1.5 (0.4; 2.6)	1.7 (0.9; 2.6)	2.1 (1.2; 3.0)	1.2 (0.2; 2.2)
Other	15.1 (12.7; 17.6)	12.5 (9.6; 15.4)	13.2 (10.9; 15.6)	17.0 (14.3; 19.8)	25.6 (19.6; 31.6)^a^
Time since immigration					
Canadian born	76.1 (73.3; 78.9)	73.4 (69.8; 77.0)	77.6 (74.6; 80.6)	76.6 (73.5; 79.7)	69.9 (64.0; 75.7)
1 or 2 years	2.3 (1.7; 2.9)	2.2 (1.2; 3.2)	1.8 (1.2; 2.4)	2.3 (1.6; 3.1)	5.4 (2.5; 8.4)^a^
3 or more years	21.6 (19.1; 24.2)	24.5 (21.1; 27.8)	20.6 (17.8; 23.3)	21.1 (18.1; 24.0)	24.7 (20.1; 29.2)
Family affluence					
Low	13.3 (11.7; 14.8)	15.2 (12.8; 17.6)	11.6 (9.9; 13.3)	13.8 (11.7; 15.9)	17.5 (13.7; 21.2)^a^
Medium	60.0 (56.7; 61.2)	61.9 (58.5; 65.2)	62.0 (59.5; 64.6)	54.1 (51.4; 56.9)	54.1 (49.4; 58.7)^a^
High	27.7 (10.5; 25.0)	22.9 (19.1; 26.8)	26.4 (23.2; 29.5)	32.1 (29.0; 35.1)	28.5 (23.3; 33.7)
Life satisfaction					
Low	17.1 (15.9; 18.2)	13.6 (11.2; 16.0)	14.9 (13.5; 16.3)	18.5 (16.7; 20.2)^a^	33.7 (29.2; 38.3)^a^
High	82.9 (81.8; 84.1)	86.4 (84,0; 88.8)	85.1 (83.7; 86.5)	81.5 (79.8; 83.3)^a^	66.3 (61.7; 70.8)^a^

^a^Significantly different proportions (95% confidence intervals that did not overlap) compared to the reference category (active social media use)

**Fig. 1:**
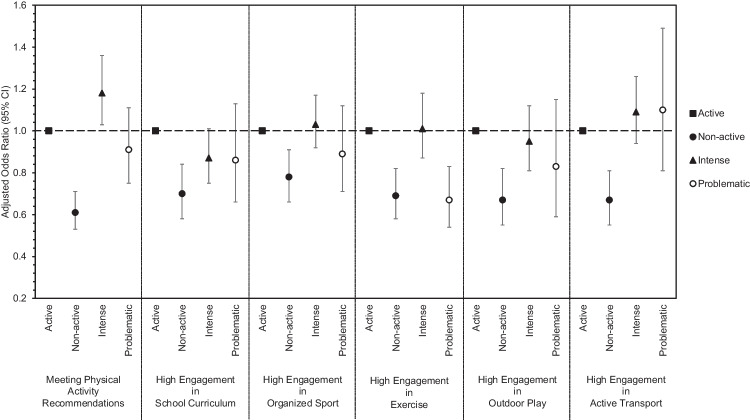
Mixed effect logistic regression models clustered by school were used to calculate the adjusted odds ratio for meeting physical activity recommendations and high engagement in each of the five physical activity domains by social media use category. Active social media use was the reference category for all models. All logistic regression models are adjusted for gender, grade, cultural and racial background, time since immigration, family affluence, and life satisfaction. The total sample size for the analysis was 12,358

The adjusted odds ratios (aOR) for the association between social media use categories and meeting physical activity recommendations and physical activity engagement in each of the five domains are presented in Fig. 1, with the detailed results provided in Supplemental Tables 2 and 3. Non-active social media use (aOR: 0.61; 95% CI: 0.53, 0.71) and intense social media use (aOR: 1.18; 95% CI: 1.03, 1.36) had statistically significant lower and higher odds, respectively, of meeting physical activity recommendations when compared to active social media use. There was no significant association in the odds of meeting physical activity recommendations for problematic social media use (aOR 0.91; 95% CI: 0.75, 1.11) when compared to active social media use. There were statistically significant lower odds of physical activity engagement across all domains for non-active social media use when compared to active social media use. Problematic social media use had a statistically significant lower odds of physical activity engagement (aOR: 0.67; 95% CI: 0.54, 0.83) in the exercise domain when compared to active social media use. The interaction terms for gender were not significant (see Supplemental Tables 4 and 5 for the stratified data). There was a marginally significant interaction for grade (see Supplemental Tables 6 and 7 for the stratified data).

## Discussion

This study aimed to investigate the association between social media use and physical activity among Canadian adolescents. We hypothesized that both non-active and problematic social media use would have lower odds of meeting physical activity recommendations. In line with our hypothesis, non-active social media use was associated with lower odds of meeting physical activity recommendations and lower odds of high engagement across all domains of physical activity. In contrast to our expectations, problematic social media use was only associated with lower odds of high physical activity engagement in the exercise domain. We also found that intense social media use was associated with a greater odds of meeting physical activity recommendations.

The theory that heightened social media use would displace time away from physical activity (i.e., the displacement theory) was not fully supported by our results. We found that intense social media use was associated with increased odds of meeting physical activity recommendations compared to active social media use, and non-active use was associated with reduced odds of meeting recommendations and engagement across all domains of physical activity. Another theory presented in literature was the *Goldilocks* theory, which is where non-active and overuse of social media is hypothesized to have a detrimental effect on adolescent health, behaviours, and well-being (Przybylski & Weinstein, 2017). Our results partially support this theory, as non-active social media use was associated with decreased odds of meeting physical activity recommendations and lower odds of high engagement in all domains of physical activity in comparison to active social media use. However, intense social media use was associated with increased odds of meeting physical activity recommendations, which was not in support of the *Goldilocks* theory. Shimoga et al. (2019) reported similar findings, where American adolescents who reported never using social media were more likely to report low levels of physical activity compared to those who used social media more frequently.

However, the findings of this study do not support that social media use displaces physical activity nor that overuse of social media is associated with lower levels of physical activity in adolescents. While we did not expect to see these results, we hypothesize that two factors, adolescent personality and social connectedness, may partially explain these associations. Adolescent personality, specifically extraversion, as defined by the Five Factor Model of Personality, was previously found to be associated with higher amounts of total physical activity and MVPA, and greater intensity and use of social media (Allen et al., 2017; Sutin et al., 2016). Factors associated with extraversion, specifically high activity level and propensity to seek out stimulation, are correlated with a more active lifestyle and less time with sedentary activities (Sutin et al., 2016). In effect, it is possible that intense social media use was indicative of an extraverted personality, which may have contributed to the significantly higher odds of meeting physical activity recommendations identified in this study.

In addition, it is possible that adolescents who do not use social media are less engaged with physical activity due to a lack of social connectedness and support from their peers. The study by Boniel-Nissim et al. (2022) supports this claim, where non-active social media use was associated with lower levels of support from both friends and classmates. It was also found that adolescent behaviours, including physical activity, are strongly related to their social networks (Haidar et al., 2019; Prochnow et al., 2020). For example, adolescents may create social connections through social media with those who share similar interests, which in turn may reinforce behaviours (Prochnow et al., 2020). The opposite may also occur, where adolescents who engage in physical activity with peers may create social connections that are maintained through social media use. Thus, our results coincide with the idea that social media may serve as a tool that reinforces physical activity habits through social connectedness. While this explains the trends seen in non-active and intense social media use, it does not explain the findings related to problematic social media use.

Similar to intense social media use, the findings of this study indicate that problematic social media use does not displace physical activity nor is it associated with reducing the odds of meeting physical activity recommendations. Previously, problematic social media use or internet addiction have been associated with lower levels of peer support and increased frequency of psychological and somatic complaints (i.e., irritability, nervousness, trouble sleeping, dizziness, and headache) (Boniel-Nissim et al., 2022; Westgate & Holliday, 2016). In addition, internet addiction, which is classified as showing addiction-like symptoms to internet use, has previously been associated with low levels of physical activity and high amounts of screen time (Han et al., 2021). As such, we hypothesized that problematic social media use would be associated with lower odds of meeting physical activity recommendations and engaging with physical activity domains. However, our results indicated that problematic social media use was only associated with lower odds of physical activity engagement in the exercise domain. It is possible that since the exercise domain is largely voluntary, being leisure-time and unscheduled physical activities, these activities are more displaceable compared to the other physical activity domains (i.e., school curriculum, organized sport, outdoor play, and active transport domains), which are largely scheduled activities (e.g., walking to school, attending club meetings, practices or games, gym class, active games at recess, or pick-up basketball) that are part of an adolescent’s daily physical activity through school.

Broadly, the findings of this study do not influence the current recommendations in the Canadian 24-Hour Movement Guidelines (Tremblay et al., 2016). While the findings of this study did not indicate a negative association of overuse of social media with physical activity levels, it does not avoid the negative effects seen on the well-being, health, and development of adolescents (Boer et al., 2022). The positive association between intense social media use and meeting physical activity recommendations is more indicative of the complexity of social behaviours, such as the affinity of personality types toward physical activity and the reinforcement of behaviours between peers, rather than the direct effect social media may have on physical activity levels of adolescents. As such, it can be theorized that other forms of screen time, such as active or passive screen time, may contribute to the negative effects seen on adolescent well-being, health, and development.

### Strengths and limitations of the study

The strengths of this study include the use of a social media scale that differentiates intensity and problematic use. This study also controlled for several potentially confounding factors that are associated with adolescent engagement in physical activity, types of physical activity, and social media use. In addition, this study had a relatively large sample size, with results weighted to represent the Canadian adolescent population. However, this study was not without limitations. First, the study incorporated a cross-sectional design which precludes the ability to identify causal relationships. Second, there was the possibility of inherent biases in the self-reported questionnaires, including self-report bias, response bias, and the propensity for adolescents to exaggerate physical activity and social media use in self-reported questionnaires (Parry et al., 2021). Third, we were unable to analyze non-binary gender groups due to a small sample size. Fourth, this study utilized a dichotomous variable for physical activity rather than a continuous measure. Last, this study excluded approximately one third of the HBSC participants as a result of missing data. We identified a difference in gender and low family affluence between the included and excluded participants. This may have masked any potential gendered differences in the analysis, including interaction terms in the logistic regression models.

## Conclusion

The findings of this study suggest that intense social media use is associated with greater odds of meeting physical activity recommendations, whereas non-active and problematic social media use were associated with lower odds of physical activity. Given the increasing prevalence of social media use among adolescents, it is important to understand both the positive and negative health impacts it may have. Thus, future research is needed to further explore these relationships to uncover the mechanisms that drive these associations. It is important to continue to find ways to harness the power of social media to help improve the health of adolescents in the future.

## Contributions to knowledge

What does this study add to existing knowledge?

- This study provided insight into the current landscape of frequency of social media use and physical activity among Canadian adolescents.
- In comparison to active social media use in Canadian adolescents, those who use it intensely had higher odds of meeting physical activity (PA) recommendations and those who do not use social media had lower odds of meeting PA recommendations and engaging in PA domains.

What are the key implications for public health interventions, practice, or policy?

- Currently, there are no guidelines for social media use that are endorsed or established by the Public Health Agency of Canada.
- This study will help inform what constitutes healthy and unhealthy use to develop or endorse current recommendations for social media use in Canadian adolescents.
- This research provides an opportunity to engage with stakeholders to address the impacts of social media use on adolescent health.

## Supplementary Information

Below is the link to the electronic supplementary material.

Supplementary file1 (DOCX 37 KB)

## Data Availability

The HBSC Canada data are available upon request by contacting Queen’s University.

## Code availability

The SAS codes used to calculate the results in this paper are available upon request by contacting the corresponding author.
