# Supplementary material for: The association between social media use and physical activity among Canadian adolescents: a Health Behaviour in School-aged Children (HBSC) study
Source: Can J Public Health. 2023 Mar 15;114(4):642–50. doi: 10.17269/s41997-023-00754-9 (PMC10349007; doi:10.17269/s41997-023-00754-9)
Supplement: Supplementary file 1 — Supplementary file1 (DOCX 37 KB) [file 41997_2023_754_MOESM1_ESM.docx]

**Supplemental Table 1.** Comparison between complete included cases to excluded incomplete cases.

| **Variable** | **Included (n= 13385)** | **Excluded (n = 8,033)** |
| --- | --- | --- |
|  | **Weighted % (95% CI)** | **Weighted % (95% CI)** |
| **Social media use category** |  |  |
| Active | 16.1 (14.7; 17.4) | 17.5 (15.6; 19.4) |
| Non-Active | 45.4 (43.7; 47.2) | 42.3 (39.6; 45.1) |
| Intense | 31.9 (29.8; 33.9) | 32.8 (29.9; 35.6) |
| Problematic | 6.7 (5.8; 7.5) | 7.5 (6.3; 8.6) |
| **Gender** |  |  |
| Boy | 45.7 (43.7; 47.7) | 51.4 (49.4; 53.4)^a^ |
| Girl | 54.3 (52.3; 56.3) | 48.6 (46.6; 50.6)^a^ |
| **Grade** |  |  |
| 6-8 | 58.2 (52.1; 64.2) | 66.0 (60.2; 71.8) |
| 9-10 | 41.8 (35.8; 47.9) | 34.0 (28.2; 39.8) |
| **Cultural and racial background** |  |  |
| White | 71.6 (66.6; 76.5) | 66.1 (59.5; 72.7) |
| Black | 3.7 (2.3; 5.1) | 7.3 (3.6; 10.9) |
| Latin American | 1.5 (1.0; 2.1) | 1.3 (0.6; 1.9) |
| East and Southeast Asian | 3.2 (1.9; 4.5) | 3.3 (2.2; 4.4) |
| East Indian and South Asian | 3.1 (1.8; 4.4) | 4.4 (2.5; 6.3) |
| Arab and West Indian | 1.8 (1.0; 2.5) | 2.0 (1.1; 3.0) |
| Other | 15.1 (12.7; 17.6) | 15.7 (13.7; 17.7) |
| **Time since immigration** |  |  |
| Canadian born | 76.1 (73.3; 78.9) | 70 (65.9; 74.1) |
| 1 or 2 years | 2.3 (1.7; 2.9) | 3.2 (2.3; 4.1) |
| 3 or more years | 21.6 (19.1; 24.2) | 26.8 (23.0; 30.6) |
| **Family affluence** |  |  |
| Low | 13.3 (11.8; 14.8) | 20.1 (17.9; 22.4)^a^ |
| Medium | 59.0 (56.7; 61.2) | 56.7 (54.3; 59.2) |
| High | 27.8 (25.0; 30.5) | 23.1 (20.4; 25.9) |
| **Life satisfaction** |  |  |
| Low | 17.1 (15.9; 18.2) | 18.4 (16.5; 20.3) |
| High | 82.9 (81.8; 84.1) | 81.6 (79.7; 83.5) |

^a^ Significantly different proportions from the included group (95% confidence intervals that did not overlap).

**Supplemental Table 2.** Unadjusted odds of meeting daily physical activity and engagement with physical activity by social media use.

| **Social media use category** | **Meeting physical activity recommendations** | **High engagement in school curriculum**** | **High engagement in organized sport**** | **High engagement in exercise**** | **High engagement in outdoor play**** | **High engagement in active transport**** |
| --- | --- | --- | --- | --- | --- | --- |
|  | **OR**  (95% CI) | **OR**  (95% CI) | **OR**  (95% CI) | **OR**  (95% CI) | **OR**  (95% CI) | **OR**  (95% CI) |
| Active (Reference) | 1.0 | 1.0 | 1.0 | 1.0 | 1.0 | 1.0 |
| Non-active | 0.66 (0.57; 0.77)* | 0.76 (0.63; 0.91)* | 0.81 (0.69; 0.95)* | 0.70 (0.60; 0.82)* | 0.82 (0.68; 1.0) | 0.69 (0.57; 0.84)* |
| Intense | 1.13 (0.99; 1.31) | 0.83 (0.72; 0.91)* | 0.99 (0.88; 1.11) | 1.01 (0.87; 1.17) | 0.88 (0.75; 1.03) | 1.07 (0.93; 1.24) |
| Problematic | 0.78 (0.64; 0.95)* | 0.74 (0.57; 0.96)* | 0.74 (0.60; 0.92)* | 0.61 (0.49; 0.76)* | 0.69 (0.51; 0.94)* | 1.07 (0.79; 1.44) |

* Significantly different proportions (95% confidence intervals that did not overlap) compared to the reference category (active social media use).

** Mean physical activity for each physical activity domain was dichotomized around the first quartile, where values below were collapsed into low engagement and values above where collapsed into high engagement.

**Supplemental Table 3.** Adjusted Odds of meeting daily physical activity and engagement with physical activity by social media use.

| **SMU Category** |  | **Meeting physical activity recommendations** | **High engagement in school curriculum**** | **High engagement in organized sport**** | **High engagement in exercise**** | **High engagement in outdoor play**** | **High engagement in active transport**** |
| --- | --- | --- | --- | --- | --- | --- | --- |
|  |  | **aOR**  (95% CI) | **aOR**  (95% CI) | **aOR**  (95% CI) | **aOR**  (95% CI) | **aOR**  (95% CI) | **aOR**  (95% CI) |
| Active (reference) |  | 1.0 | 1.0 | 1.0 | 1.0 | 1.0 | 1.0 |
| Non-active |  | 0.61 (0.53; 0.71)* | 0.70 (0.58; 0.84)* | 0.75 (0.63; 0.88)* | 0.69 (0.58; 0.82)* | 0.67 (0.55; 0.82)* | 0.67 (0.55; 0.81)* |
| Intense |  | 1.18 (1.03; 1.36)* | 0.87 (0.75; 1.01) | 1.03 (0.91; 1.16) | 1.01 (0.87; 1.18) | 0.95 (0.81; 1.12) | 1.09 (0.94; 1.26) |
| Problematic |  | 0.91 (0.75; 1.11) | 0.86 (0.66; 1.13) | 0.88 (0.70; 1.10) | 0.67 (0.54; 0.83)* | 0.83 (0.59; 1.15) | 1.10 (0.81; 1.49) |
| **Predictors** |  |  |  |  |  |  |  |
| **Variable** | **Reference Category** | **Estimate**  (95% CI) | **Estimate**  (95% CI) | **Estimate**  (95% CI) | **Estimate**  (95% CI) | **Estimate**  (95% CI) | **Estimate**  (95% CI) |
| Gender | Male | 0.63 (0.55; 0.71)* | 0.75 (0.65; 0.86)* | 1.0 (0.89; 1.12) | 0.88 (0.76; 1.03) | 0.64 (0.56; 0.73)* | 0.84 (0.73; 0.98)* |
| Grade | 6-8 | 0.83 (0.71; 0.96)* | 0.80 (0.65; 0.98)* | 0.68 (0.59; 0.77)* | 0.92 (0.80; 1.07) | 0.39 (0.32; 0.46)* | 0.90 (0.76; 1.05) |
| Cultural and Racial Background | White | Black:  0.76 (0.57; 1.01)  Latin American:  0.85 (0.55; 1.30)  East and Southeast Asian:  0.77 (0.55; 1.07)  East Indian and South Asian:  0.97 (0.72; 1.31)  Arab and West  Indian:  0.71 (0.45; 1.12)  Other:  1.01 (0.85; 1.19) | Black:  0.90 (0.56; 1.45)  Latin American:  0.83 (0.53; 1.31)  East and Southeast Asian:  1.05 (0.72; 1.54)  East Indian and South Asian:  0.75 (0.54; 1.05)  Arab and West  Indian:  0.61 (0.43; 0.87)*  Other:  0.86 (0.72; 1.02) | Black:  0.74 (0.57; 0.96)*  Latin American:  0.74 (0.50; 1.10)  East and Southeast Asian:  0.64 (0.48; 0.85)*  East Indian and South Asian:  0.55 (0.43; 0.71)*  Arab and West  Indian:  0.57 (0.40; 0.80)*  Other:  0.84 (0.72; 0.98)* | Black:  0.74 (0.56; 0.99)*  Latin American:  0.70 (0.46; 1.09)  East and Southeast Asian:  0.64 (0.48; 0.85)*  East Indian and South Asian:  0.80 (0.60; 1.08)  Arab and West  Indian:  0.85 (0.53; 1.36)  Other:  0.96 (0.80; 1.14) | Black:  0.52 (0.34; 0.81)*  Latin American:  0.66 (0.36; 1.23)  East and Southeast Asian:  0.51 (0.35; 0.76)*  East Indian and South Asian:  1.13 (0.72; 1.78)  Arab and West  Indian:  0.72 (0.45; 1.15)  Other:  0.95 (0.76; 1.18) | Black:  0.63 (0.42; 0.95)*  Latin American:  0.81 (0.43; 1.50)  East and Southeast Asian:  1.03 (0.67; 1.60)  East Indian and South Asian:  1.08 (0.70; 1.60)  Arab and West  Indian:  0.80 (0.43; 1.47)  Other:  1.13 (0.90; 1.42) |
| Time Since Immigration | Canadian born | 1-2 years:  0.66 (0.48; 0.91)*  3+ years:  0.84 (0.73; 0.97)* | 1-2 years:  0.54 (0.37; 0.80)*  3+ years:  0.93 (0.82; 1.07) | 1-2 years:  0.54 (0.38; 0.77)*  3+ years:  0.92 (0.80; 1.06) | 1-2 years:  1.03 (0.73; 1.45)  3+ years:  1.01 (0.87; 1.17) | 1-2 years:  1.21 (0.75; 1.95)  3+ years:  0.84 (0.71; 0.98)* | 1-2 years:  1.02 (0.56; 1.87)  3+ years:  1.07 (0.88; 1.29) |
| FAS | Low | Medium:  1.20 (1.03; 1.39)*  High:  1.90 (1.58; 2.28)* | Medium:  1.83 (1.58; 2.12)*  High:  3.35 (2.74; 4.10)* | Medium:  1.78 (1.54; 2.05)*  High:  3.22 (2.66; 3.89)* | Medium:  1.23 (1.01; 1.50)*  High:  2.06 (1.67; 2.56)* | Medium:  1.17 (0.96; 1.42)  High:  1.61 (1.25; 2.07)* | Medium:  0.91 (0.70; 1.18)  High:  0.96 (0.75; 1.23) |
| Life Satisfaction | Low | 1.58 (1.34; 1.85)* | 1.75 (1.51; 2.02)* | 1.75 (1.52; 2.01)* | 1.42 (1.21; 1.68)* | 1.44 (1.20; 1.74)* | 1.12 (0.95; 1.33) |

* Significantly different proportions (95% confidence intervals that did not overlap) compared to the reference category (active social media use).

** Mean physical activity for each physical activity domain was dichotomized around the first quartile, where values below were collapsed into low engagement and values above where collapsed into medium to high engagement.

**Supplemental Table 4.** Adjusted odds of meeting daily physical activity and engagement with physical activity by social media use for boys.

| **Social media use category** | **Meeting physical activity recommendations** | **High engagement in school curriculum**** | **High engagement in organized sport**** | **High engagement in exercise**** | **High engagement in outdoor play**** | **High engagement in active transport**** |
| --- | --- | --- | --- | --- | --- | --- |
|  | **OR**  (95% CI) | **OR**  (95% CI) | **OR**  (95% CI) | **OR**  (95% CI) | **OR**  (95% CI) | **OR**  (95% CI) |
| Active (Reference) | 1.0 | 1.0 | 1.0 | 1.0 | 1.0 | 1.0 |
| Non-active | 0.65 (0.53; 0.80)* | 0.72 (0.56; 0.92)* | 0.71 (0.56; 0.89)* | 0.68 (0.54; 0.86)* | 0.61 (0.45; 0.82)* | 0.67 (0.50; 0.90)* |
| Intense | 1.17 (0.96; 1.43) | 0.87 (0.69; 1.10) | 0.93 (0.78; 1.11) | 1.01 (0.81; 1.26) | 1.02 (0.77; 1.36) | 1.00 (0.79; 1.27) |
| Problematic | 0.86 (0.62; 1.20) | 1.10 (0.68; 1.77) | 0.75 (0.53; 1.06) | 0.67 (0.45; 0.99)* | 0.87 (0.53; 1.40) | 0.96 (0.62; 1.50) |

* Significantly different proportions (95% confidence intervals that did not overlap) compared to the reference category (active social media use).

** Mean physical activity for each physical activity domain was dichotomized around the first quartile, where values below were collapsed into low engagement and values above where collapsed into high engagement.

**Supplemental Table 5.** Adjusted odds of meeting daily physical activity and engagement with physical activity by social media use for girls.

| **Social media use category** | **Meeting physical activity recommendations** | **High engagement in school curriculum**** | **High engagement in organized sport**** | **High engagement in exercise**** | **High engagement in outdoor play**** | **High engagement in active transport**** |
| --- | --- | --- | --- | --- | --- | --- |
|  | **OR**  (95% CI) | **OR**  (95% CI) | **OR**  (95% CI) | **OR**  (95% CI) | **OR**  (95% CI) | **OR**  (95% CI) |
| Active (Reference) | 1.0 | 1.0 | 1.0 | 1.0 | 1.0 | 1.0 |
| Non-active | 0.57 (0.46; 0.69)* | 0.70 (0.53; 0.92)* | 0.78 (0.63; 0.97)* | 0.69 (0.54; 0.89)* | 0.74 (0.59; 0.94)* | 0.65 (0.52; 0.81)* |
| Intense | 1.18 (0.97; 1.43) | 0.87 (0.73; 1.04) | 1.10 (0.93; 1.28) | 1.00 (0.83; 1.20) | 0.92 (0.76; 1.11) | 1.13 (0.92; 1.39) |
| Problematic | 0.93 (0.72; 1.20) | 0.78 (0.56; 1.09) | 0.97 (0.74; 1.29) | 0.64 (0.49; 0.83)* | 0.81 (0.53; 1.22) | 1.17 (0.78; 1.76) |

* Significantly different proportions (95% confidence intervals that did not overlap) compared to the reference category (active social media use).

** Mean physical activity for each physical activity domain was dichotomized around the first quartile, where values below were collapsed into low engagement and values above where collapsed into high engagement.

**Supplemental Table 6.** Adjusted odds of meeting daily physical activity and engagement with physical activity by social media use for grades 6 to 8.

| **Social media use category** | **Meeting physical activity recommendations** | **High engagement in school curriculum**** | **High engagement in organized sport**** | **High engagement in exercise**** | **High engagement in outdoor play**** | **High engagement in active transport**** |
| --- | --- | --- | --- | --- | --- | --- |
|  | **OR**  (95% CI) | **OR**  (95% CI) | **OR**  (95% CI) | **OR**  (95% CI) | **OR**  (95% CI) | **OR**  (95% CI) |
| Active (Reference) | 1.0 | 1.0 | 1.0 | 1.0 | 1.0 | 1.0 |
| Non-active | 0.60 (0.50; 0.73)* | 0.67 (0.52; 0.85)* | 0.76 (0.62; 0.93)* | 0.76 (0.62; 0.94)* | 0.71 (0.53; 0.94)* | 0.70 (0.55; 0.90)* |
| Intense | 1.04 (0.89; 1.22) | 0.78 (0.63; 0.96)* | 0.99 (0.82; 1.19) | 1.01 (0.82; 1.24) | 0.81 (0.65; 1.02) | 1.06 (0.85; 1.31) |
| Problematic | 0.86 (0.65; 1.15) | 0.86 (0.57; 1.30) | 0.93 (0.67; 1.28) | 0.80 (0.57; 1.13) | 0.65 (0.42; 1.00) | 0.86 (0.55; 1.35) |

* Significantly different proportions (95% confidence intervals that did not overlap) compared to the reference category (active social media use).

** Mean physical activity for each physical activity domain was dichotomized around the first quartile, where values below were collapsed into low engagement and values above where collapsed into high engagement.

**Supplemental Table 7.** Adjusted odds of meeting daily physical activity and engagement with physical activity by social media use for grades 9 and 10.

| **Social media use category** | **Meeting physical activity recommendations** | **High engagement in school curriculum**** | **High engagement in organized sport**** | **High engagement in exercise**** | **High engagement in outdoor play**** | **High engagement in active transport**** |
| --- | --- | --- | --- | --- | --- | --- |
|  | **OR**  (95% CI) | **OR**  (95% CI) | **OR**  (95% CI) | **OR**  (95% CI) | **OR**  (95% CI) | **OR**  (95% CI) |
| Active (Reference) | 1.0 | 1.0 | 1.0 | 1.0 | 1.0 | 1.0 |
| Non-active | 0.58 (0.45; 0.75)* | 0.75 (0.57; 1.00)* | 0.69 (0.52; 0.91)* | 0.54 (0.40; 0.73)* | 0.58 (0.45; 0.76)* | 0.57 (0.41; 0.80)* |
| Intense | 1.38 (1.14; 1.66)* | 0.99 (0.81; 1.21) | 1.07 (0.90; 1.27) | 0.99 (0.80; 1.22) | 1.06 (0.85; 1.31) | 1.13 (0.90; 1.41) |
| Problematic | 0.96 (0.71; 1.30) | 0.89 (0.61; 1.30) | 0.85 (0.62; 1.17) | 0.54 (0.41; 0.73)* | 1.00 (0.63; 1.58) | 1.44 (0.92; 2.25) |

* Significantly different proportions (95% confidence intervals that did not overlap) compared to the reference category (active social media use).

** Mean physical activity for each physical activity domain was dichotomized around the first quartile, where values below were collapsed into low engagement and values above where collapsed into high engagement.
